# Phytochemical characterization and biological properties of two standardized extracts from a non‐psychotropic *Cannabis sativa* L. cannabidiol (CBD)‐chemotype

**DOI:** 10.1002/ptr.7201

**Published:** 2021-06-26

**Authors:** Claudia Muscarà, Antonella Smeriglio, Domenico Trombetta, Giuseppina Mandalari, Erminia La Camera, Gianpaolo Grassi, Clara Circosta

**Affiliations:** ^1^ Department of Chemical, Biological, Pharmaceutical and Environmental Sciences University of Messina Messina Italy; ^2^ Foundation Prof. Antonio Imbesi Messina Italy; ^3^ Council for Agricultural Research and Economics Research Centre for Cereal and Industrial Crops (CREA‐CI) IT Rovigo Italy

**Keywords:** antimicrobial, antioxidant, cannabidiol, *Cannabis sativa* L., non‐psychotropic cannabinoids, standardized extracts

## Abstract

The aim of study was to evaluate and compare the phytochemical profile, the antioxidant and antimicrobial properties of two standardized extracts from non‐psychotropic (Δ^9^‐tetrahydrocannabinol ≤0.2%) *Cannabis sativa* L. var. *fibrante* rich in cannabidiol (CBD). The two extracts, namely Cannabis Fibrante Hexane Extract 1 (CFHE1) and Cannabis Fibrante Hexane Extract 2 (CFHE2), were obtained by extraction with acidified hexane from dried flowering tops as such and after hydrodistillation of the essential oil, respectively. Gas chromatographic analysis showed that cannabinoids remained the predominant class of compounds in both extracts (82.56% and 86.38%, respectively), whereas a marked depletion of the terpenes occurred. Moreover, liquid chromatographic analysis highlighted a high titer of cannabidiol acid (CBDA) and CBD in CFHE1 and CFHE2, respectively. Both extracts showed a strong and concentration‐dependent antioxidant activity and a potent antimicrobial activity against both *Staphylococcus aureus* ATCC 6538 (MIC and MBC of 4.88 μg/ml for CFHE1, and 4.88 and 19.53 μg/ml, respectively, for CFHE2) and methicillin resistant clinical strains (MIC values between 1.22 and 9.77 μg/ml and MBC values between 4.88 and 78.13 μg/ml). Considering this, the obtained results suggest that standardized extracts of *C. sativa* var. *fibrante* could find promising applications as novel antimicrobial agents.

## 1 INTRODUCTION

*Cannabis sativa* L., belonging to the *Cannabaceae* family, is a well‐known dioicous plant, since it is among the most used and cultivated plants worldwide, due to its strong ability to adapt to various pedoclimatic conditions, which allowed its extensive geographical distribution. *Cannabis sativa* has a wide range of therapeutic applications against several diseases (Novack, 2016; Russo, 2017), but it is also used for food purposes as a source of nutrients and non‐nutrient compounds (Callaway, 2004; Kaul et al., 2008; Prociuk et al., 2008; Smeriglio et al., 2016) and as ecological raw material, finding applications in the textile industry and bioengineering (Mutje, Lopez, Vallejos, Lopez, & Vilaseca, 2007; Westerhuis, 2016).

In the past, the taxonomic classification of *Cannabis* has been complicated due to its genetic variability. Recently, it has been recognized as monotypic genus including only the species *C. sativa*; however, it can be differentiated into different chemotypes depending on the cannabinoid profile (de Meijer, 2014). Compared to the drug‐type, fiber‐type *C. sativa* is characterized by a low Δ^9^‐tetrahydrocannabinol (Δ^9^‐THC, <0.2%) content with respect to the other non‐psychoactive cannabinoids. The marked presence of a cannabinoid compared to the others determines the *Cannabis* chemotype, such as cannabidiol (CBD), cannabigerol (CBG) and cannabidivarin chemotypes (Muscarà et al., 2021; Smeriglio et al., 2018). Considering this, despite *C. sativa* represents an interesting crop for several industrial uses, both the European and U.S. legislation require a strict control of cannabinoids type and content for cultivation and subsidies release (Pacifico et al., 2006).

Due to the psychotropic effects of Δ^9^‐THC, research has recently focused almost exclusively on low‐THC fiber chemotypes, also evaluating other previously neglected secondary metabolites such as polyphenols and terpenes. Terpenes, in particular, having a common precursor (geranyl pyrophosphate) with cannabinoids, produced through the deoxyxylulose pathway (Fellermeier, Eisenreich, Bacher, & Zenk, 2001) could act synergistically with them in carrying out the observed biological activities (Iseppi et al., 2019; Pellati et al., 2018; Rupasinghe, Davis, Kumar, Murray, & Zheljazkov, 2020; Russo, 2011; Smeriglio et al., 2018; Smeriglio et al., 2020). Among the non‐psychoactive phytocannabinoids, CBD represents the most promising from pharmaceutical point of view, due to its several beneficial effects. CDB has been shown to possess antioxidant, anti‐inflammatory and antimicrobial activity, as well as anxiolytic, neuroprotective and anticonvulsant properties (Appendino et al., 2008; Esposito et al., 2007; Pagano et al., 2016; Szaflarski et al., 2018; Zuardi, de Souza Crippa, Hallak, Campos, & Guimarães, 2017). These data have been supported by many studies on *Cannabis* plant complexes, including extracts and essential oils rich in this cannabinoid (Carvalho et al., 2020; Gabotti et al., 2019; Iseppi et al., 2019; Smeriglio et al., 2018; Smeriglio et al., 2020).

Current available studies on *Cannabis* extracts are still rather lacking and mainly focused on phytochemical features. Moreover, most of these have used non‐standardized extracts, and this aspect has a critical impact in order to ensure the reproducibility of the observed biological effects.

Considering this, the aim of study was to evaluate and compare, for the first time, the phytochemical profile as well as the antioxidant and antimicrobial properties of two different standardized extracts obtained from dried flowering tops (as such and after hydrodistillation of the essential oil) of a non‐psychotropic CBD‐rich *C. sativa* L. var. *fibrante*.

## 2 MATERIALS AND METHODS

### 2.1 Chemical reagents

All chemicals were of analytical grade and were purchased from Sigma‐Aldrich (Milan, Italy). Liquid chromatography (LC) and gas chromatography (GC)‐grade solvents were purchased from Merck (Darmstadt, Germany). Certified reference standard solutions (Cerilliant®) of CBD, cannabinol (CBN) and Δ^9^‐tetrahydrocannabinolic acid A (THCAA) were purchased from Merck (Darmstadt, Germany). The reference standard of cannabidiolic acid (CBDA) was purchased from Restek (Milan, Italy).

### 2.2 Plant material and sample preparation

Dried flowering tops of *C. sativa* L. var. *fibrante* were provided by the Council for Agricultural Research and Agricultural Economy Analysis – Research Center for Industrial Crops (CREA‐CI, Rovigo, Italy). A voucher specimen (19/05 CSF) was deposited at the Department ChiBioFarAm, University of Messina (Messina, Italy). Sample preparation was carried out according to Smeriglio et al. (2018). *Cannabis* dried flowering tops as such and after hydrodistillation of the essential oil (Smeriglio et al., 2020), were extracted in order to obtain two hexane extracts namely Cannabis Fibrante Hexane Extract 1 (CFHE1) and Cannabis Fibrante Hexane Extract 2 (CFHE2), respectively. Fifty (50) grams of dried flowering tops were extracted three times with 500 ml of 0.1% acetic acid/hexane (*v/v*), sonicating for 5 min, and proceeding the extraction under constant agitation for 3 hr at room temperature (RT), in the dark. Finally, the three sequential extracts were combined and dried by a rotary evaporator (Buchi R‐205, Cornaredo, Italy). The dry extracts (DEs) were stored in dark sealed vials with nitrogen headspace at −20°C until analysis.

### 2.3 Total phenols assay

Total phenols were determined by Folin–Ciocalteu method as described by Smeriglio et al. (2016) using gallic acid as reference compound (2.5–20 μg/ml). Briefly, 500 μl of Folin–Ciocalteu reagent, 450 μl of deionization water and 50 μl of CFHE 1 or CFHE2 (4.2–33.3 μg/ml) were mixed, and after 3 min 500 μl of Na_2_CO_3_ 10% (*w/v*) was added to the reaction mixture. Samples were subjected to 1 hr of incubation at RT, in the dark, mixing every 10 min and then the absorbance was recorded at 786 nm. Total phenols were expressed as mg of gallic acid equivalents (GAE)/100 g of DE.

### 2.4 Phytochemical characterization by gas chromatography–mass spectrometry analysis

Gas chromatography–mass spectrometry (GC–MS) analysis was carried out by an Agilent 7890A gas chromatograph equipped with an Agilent 5975C mass spectrometry detector. Elution was performed using an Agilent HP‐5MS column (30 mm, 0.25 mm, 0.25 μm) according to the method reported and validated by Smeriglio et al. (2020), recording the mass spectra in the 40–400 *m/z* range. The identification was carried out considering the retention index calculated with respect to the C_7_‐C_40_ *n*‐alkanes mix on the HP‐5MS column, comparing the mass spectra and MS fragmentation patterns with MS data of NIST08 library and with those reported in the literature (Adams, 2007) as well as by co‐injection with reference standards (α‐bisabolol, α‐caryophyllene, α‐pinene, β‐caryophyllene, β‐pinene, caryophyllene oxide, limonene, and CBD).

### 2.5 Phytocannabinoid profile by reversed‐phase liquid chromatography coupled with diode array detection and electrospray ion trap tandem mass spectrometry analysis

The quali‐quantitative analysis of main acid and neutral phytocannabinoids was carried out using an Agilent high‐performance LC system (1100 series) equipped with a diode‐array (DAD) (G1315) and an ion trap mass spectrometer detector (6320). Electrospray ion (ESI) source operating both in positive and in negative ionization mode was chosen because acid cannabinoids ionize better in negative ionization‐mode, while neutral cannabinoids show a higher signal in positive ionization‐mode. The chromatographic separation was performed on a Luna Omega PS C18 (150 × 2.1 mm, 5 μm; Phenomenex) with solvent A (0.1% formic acid) and solvent B (acetonitrile). The elution program was the following: 0–6 min, 50% B; 6–12 min, 57% B; 12–22 min, 57% B; 22–23 min, 50% B; 23–25 min, 50% B. The flow rate was 0.4 ml/min, whereas the column temperature and the injection volume were 28°C and 5 μl, respectively. UV–Vis spectra of phytocannabinoids were recorded in the range 190–600 nm and chromatograms were acquired at 220 nm. Nitrogen was employed as dry gas in mass spectrometry with a flow rate set at 10 L/min, 32 psi and 350°C, according to Pellati et al. (2018). Capillary and skimmer voltage were 3.5 kV and 40 V, respectively. Data acquisition was performed in full‐scan mode within the scan range 90–1,000 *m/z*. Data processing was carried out by Agilent 6300 Series Ion Trap LC/MS system software (version 6.2). To confirm the identified peaks, the retention time, mass and UV–Vis spectra were compared with literature data and with reference standards (CBD, CBN, THCAA, and CBDA). The quantification of phytocannabinoids was performed by built external standard calibration curves with reference compounds and results were expressed as mg of each cannabinoid/100g DE. Regarding phytocannabinoids for which reference standards were not currently available, the quantification was carried out by using the calibration curve of the most structurally similar cannabinoid. In particular, the reference compound CBN was used both for its acid form cannabinolic acid (CBNA) and for its degradation product cannabicyclol (CBL).

### 2.6 Antioxidant and free‐radical scavenging activity

The antioxidant and free‐radical scavenging activities of CFHE1 and CFHE2 were determined spectrophotometrically by colorimetric in vitro cell‐free assays, which differ in the reaction mechanisms and environments. Absorbance data recorded by an UV–VIS Spectrophotometer (Shimadzu UV‐1601), were expressed as half‐inhibitory concentration (IC_50_, μg/ml) with confident limits (C.L.) at 95% by Litchfield and Wilcoxon test, using the PHARM/PCS software version 4 (MCS Consulting, Wynnewood, PA). A preliminary screening was carried out to select the optimal concentration range for samples and reference compounds. The concentration ranges reported below refer to the final concentrations of the samples or reference standards in the reaction mixture. Sample solutions, colorless at the tested concentrations, did not show any interference in the colorimetric tests performed.

### 2.7 Trolox equivalent antioxidant capacity

The scavenging capacity of CFHE1 and CFHE2 against ABTS^•+^ was carried out according to Smeriglio, Mandalari, et al. (2016). Briefly, trolox equivalent antioxidant capacity (TEAC) assay was performed using a reaction mixture consisting of 1.7 mM ABTS and 4.3 mM potassium persulfate 5:1 (*v/v*), left at RT in the dark for at least 12 hr, and then used between 12 and 16 hr after preparation. Before use, reaction mixture was diluted with phosphate buffer (pH 7.4) in order to obtain an absorbance of 0.7 ± 0.02 at 734 nm. Fifty microliters of sample (CFHE1 and CFHE2, 1.50–12.0 μg/ml), reference compound (trolox, 0.625–5.0 μg/ml) or blank (hexane) were added to 1 ml of diluted reaction mixture, and after 6 min of incubation at RT in the dark, the absorbance was recorded at 734 nm.

### 2.8 Oxygen radical absorbance capacity

The antioxidant capacity of CFHE1 and CFHE2 against 2,2‐azobis(2‐amidinopropane)‐dihydrochloride (AAPH) peroxyl radical was carried out according to Barreca et al. (2016). Briefly, oxygen radical absorbance capacity (ORAC) assay was performed by mixing 20 μl of sample solution (0.75–6.0 μg/ml and 2.5–20.0 μg/ml for CFHE1 and CFHE2, respectively), standard (trolox, 0.25–2.5 μg/ml) or 75 mM phosphate buffer (pH 7.4) with 120 μl of 117 nM fluorescein. After 15 min of pre‐incubation at 37°C, 60 μl of fresh 40 mM AAPH solution were added. Fluorescence was recorded every 30 s for 90 min (λ_ex_ 485; λ_em_ 520) using a Fluorescence Plate Reader (FLUOStar Omega, BMG LABTECH).

### 2.9 β‐Carotene bleaching

The β‐carotene bleaching assay was carried out according to Smeriglio et al. (2017). A β‐carotene emulsion was prepared by mixing β‐carotene chloroform solution (1 mg/ml), 40 μl of linoleic acid and 400 μl of Tween‐40. After removing the chloroform with the rotary evaporator (Buchi R‐205, Cornaredo Italy), the film was resuspended with 50 ml of pre‐oxygenated water. An emulsion prepared in the same conditions but without β‐carotene was used as negative control. After that, 8 ml of β‐carotene emulsion were aliquoted in borosilicate tubes and 320 μl of each sample solution (5–40 μg/ml for CFHE1 and CFHE2), reference standard (butylated hydroxytoluene, 0.031–0.25 μg/ml) or blank (hexane) were added and incubated in the dark at 50°C in a water bath. The absorbance was recorded at the starting time (T0) and every 20 min until 120 min at 470 nm.

### 2.10 Iron‐chelating activity

Iron‐chelating activity was evaluated by ferrozine assay as described by Smeriglio et al. (2017). Fifty microliters of CFHE1 or CFHE2 sample solution (10.0–80.0 μg/ml), reference standard (ethylenediaminetetraacetic acid, 1.5–12.0 μg/ml) or blank (hexane) were added to 25 μl of 2 mM FeCl_2_ • 4H_2_O and incubated at RT for 5 min. After that, 50 μl of 5 mM ferrozine and 1,375 μl of deionized water were added to the reaction mixture. The absorbance was recorded after 10 min at 562 nm.

### 2.11 Ferric reducing antioxidant power

The antioxidant activity of CFHE1 and CFHE2 against the 2,4,6,‐Tris(2‐pyridyl)‐s‐triazine (TPTZ) radical was carried out as described by Smeriglio, Mandalari, et al. (2016). Ferric reducing antioxidant power (FRAP) reagent was prepared by mixing (1:1:10, *v/v/v*) three pre‐heated (10 min at 37°C) reagent solutions: 10 mM TPTZ‐40 mM HCl solution, 20 mM FeCl_3_ and 300 mM buffer acetate pH 3.6.

Fifty (50) microliters of sample solution (25–200 μg/ml for CFHE1 and CFHE2), reference compound (trolox, 1.25–10 μg/ml) or blank (hexane) were added to 1 ml of FRAP reagent, and after 4‐min incubation at RT, the absorbance was recorded at 593 nm.

### 2.12 2,2‐diphenyl‐1‐picrylhydrazyl assay

The radical scavenging activity of CFHE1 and CFHE2 was evaluated by 2,2‐diphenyl‐1‐picrylhydrazyl (DPPH) assay as described by Smeriglio et al. (2017). Briefly, 37.5 μl of sample solution (50–400 μg/ml for both CFHE1 and CFHE2), reference standard (trolox, 1.25–10 μg/ml) or blank (hexane) were added to 1,500 μl of 10^−4^ M DPPH methanol solution and incubated in the dark at RT for 20 min. The absorbance was recorded at 517 nm.

### 2.13 Antimicrobial activity

The following strains were used for the antimicrobial assays: *Staphylococcus aureus* ATCC 6538, 19 methicillin‐resistant *S. aureus* (MRSA) clinical strains of *S. aureus* (identified with serial number from 1 to 19) obtained from orthopedic sites (La Camera et al., 2018), *Escherichia coli* ATCC 10536, *Pseudomonas aeruginosa* ATCC 9027 and *Candida albicans* ATCC 10231.

The minimum inhibitory concentration (MIC), the minimum fungicidal concentration (MFC), and the minimum bactericidal concentration (MBC) of CFHE1 or CFHE2 were determined by the broth microdilution method, according to Clinical and Laboratory Standards Institute methods (CLSI 2008; 2012). The tested concentrations ranged from 1.22 to 2,500 μg/ml of either CFHE1 or CFHE2 dissolved in DMSO. The final concentration of DMSO did not exceed 1% in each sample. Positive controls with antibiotics (vancomycin for the *S. aureus* strains and tobramycin for the Gram‐negative strains) and antifungals (caspofungin) were used. A control with the extract alone was also included in each assay to check and avoid any interference at the concentrations tested. The MIC was defined as the lowest concentration, which completely inhibited bacterial growth after 20 hr. The MFC was defined as the lowest concentration, which completely inhibited fungal growth after 48 hr. The MBCs were determined by seeding 20 μl from all clear MIC wells onto Mueller–Hinton agar (Oxoid) plates. The MBC was defined as the lowest extract concentration that killed 99.9% of the final inocula after 24‐hr incubation.

### 2.14 Statistical analysis

Results were expressed as mean ± SD of three independent experiments in triplicate (*n* = 3). Data were analyzed by one‐way analysis of variance (ANOVA) followed by Dunnett's test for antioxidant and antimicrobial assays, and Tukey's test for chemical characterization by SigmaPlot 12.0 software (Systat Software Inc., San Jose, CA). Results were considered statistically significant for *p* < .05.

## 3 RESULTS AND DISCUSSION

### 3.1 Phytochemical analyses

The sample preparation and extraction method adopted in the present study allowed to obtain the following extraction yields: 5.82% and 7.08% for the extracts obtained from dried flowering tops as such (CFHE1) and after hydrodistillation of the essential oil (CFHE2), respectively. A preliminary phytochemical screening by Folin–Ciocalteu method highlighted a high total phenol content in both extracts: 19,108 mg GAE/100 g DE and 8,587 mg GAE/100 g DE for CFHE1 and CFHE2, respectively. GC–MS analysis led to the identification of 83 and 48 compounds in CFHE1 and CFHE2, respectively (Table 1).

**TABLE 1:** Phytochemical profile of hexane extracts (CFHE1 and CFHE2) of *Cannabis sativa* var. *fibrante* by GC–MS analysis. Results were expressed as mean area percentage (%) ± SD of three independent experiments in triplicate (*n* = 3)

#	KI^a^	Compound	CFHE1	CFHE2
1	902	Heptanal	0.01 ± 0.00^*^	—
2	930	α‐Thujene	*t*	—
3	939	α‐Pinene	0.06 ± 0.00^*^	—
4	954	Camphene	*t*	—
5	973	Hexanoic acid	0.02 ± 0.00^*^	0.01 ± 0.00
6	979	β‐Pinene	0.02 ± 0.00^*^	—
7	988	Myrcene	0.04 ± 0.00^*^	—
8	1,011	δ‐3‐Carene	*t*	—
9	1,017	α‐Terpinene	—	0.01 ± 0.00^§^
10	1,029	Limonene	0.06 ± 0.00^*^	—
11	1,031	1,8‐cineole	0.03 ± 0.00^*^	—
12	1,059	γ‐Terpinene	0.01 ± 0.00	0.01 ± 0.00
13	1,070	cis‐Sabinene hydrate	0.01 ± 0.00^*^	—
14	1,074	Heptanoic acid	0.01 ± 0.00^*^	—
15	1,088	Terpinolene	0.01 ± 0.00^*^	—
16	1,098	Linalool	0.01 ± 0.00^*^	—
17	1,116	Fenchol	0.04 ± 0.00^*^	—
18	1,122	trans‐ρ‐Mentha‐2,8‐dien‐1‐ol	—	0.01 ± 0.00^§^
19	1,169	Borneol	0.04 ± 0.00	0.04 ± 0.00
20	1,188	α‐Terpineol	0.04 ± 0.00^*^	—
21	1,189	1‐Dodecene	*t*	*t*
22	1,270	Nonanoic acid	0.02 ± 0.00^*^	—
23	1,311	6‐hydroxy‐Carvotanacetone	*t*	—
24	1,367	n‐Undecanol	—	0.02 ± 0.00^§^
25	1,371	Ciclosativene	0.01 ± 0.00^*^	—
26	1,375	α‐Ylangene	0.02 ± 0.00^*^	—
27	1,376	α‐Copaene	0.01 ± 0.00^*^	—
28	1,382	β‐Panasinsene	0.01 ± 0.00^*^	—
29	1,408	(Z)‐β‐Caryophyllene	0.01 ± 0.00^*^	—
30	1,409	α‐Gurjunene	0.01 ± 0.00^*^	—
31	1,417	(E)‐β‐Caryophyllene	2.26 ± 0.05^*^	0.02 ± 0.00
32	1,434	α‐trans‐Bergamotene	0.01 ± 0.00^*^	—
33	1,441	Aromadendrene	0.01 ± 0.00^*^	—
34	1,456	α‐Caryophyllene	0.01 ± 0.00	0.01 ± 0.00
35	1,458	(E)‐β‐Farnesene	0.02 ± 0.00^*^	—
36	1,460	Allo‐Aromadendrene	0.05 ± 0.00^*^	—
37	1,466	9‐epi‐(E)‐Caryophyllene	0.01 ± 0.00^*^	—
38	1,477	γ‐Gurjunene	0.16 ± 0.01^*^	—
39	1,484	α‐Amorphene	0.01 ± 0.00^*^	—
40	1,490	β‐Selinene	0.11 ± 0.01^*^	*t*
41	1,492	δ‐Selinene	0.04 ± 0.01^*^	—
42	1,496	Valencene	0.02 ± 0.00^*^	—
43	1,497	Viridiflorene	0.08 ± 0.00^*^	—
44	1,502	α‐Muurolene	*t*	—
45	1,505	(E,E)‐α‐Farnesene	0.01 ± 0.00^*^	*t*
46	1,513	γ‐Cadinene	0.03 ± 0.00^*^	0.01 ± 0.00
47	1,515	(Z)‐γ‐Bisabolene	0.02 ± 0.00^*^	—
48	1,526	δ‐Cadinene	0.03 ± 0.00^*^	—
49	1,535	Dihydroactinolide	—	0.03 ± 0.00^§^
50	1,546	Selina‐3,7(11)‐diene	0.02 ± 0.00^*^	—
51	1,563	(E)‐Nerolidol	0.07 ± 0.00^*^	—
52	1,566	Dodecanoic acid	—	0.04 ± 0.00^§^
53	1,572	Caryophyllene alcohol	0.05 ± 0.00^*^	—
54	1,583	Caryophyllene oxide	1.46 ± 0.12^*^	0.03 ± 0.00
55	1,602	Ledol	0.01 ± 0.00^*^	—
56	1,627	Benzophenone	—	0.01 ± 0.00^§^
57	1,640	Caryophylla −4(12),8(13)‐dien‐5β‐ol	0.34 ± 0.02^*^	—
58	1,685	α‐Bisabolol	0.04 ± 0.00^*^	*t*
59	1,698	Loliolide	0.02 ± 0.00^*^	—
60	1700	Heptadecane	0.06 ± 0.00	0.06 ± 0.00
61	1702	Eudesm‐7(11)‐en‐4‐ol	0.01 ± 0.00^*^	—
62	1715	(2E,6Z)‐Farnesol	—	0.05 ± 0.00^§^
63	1723	Tetradecanoic acid	0.38 ± 0.03^*^	0.25 ± 0.01
64	1758	3,6‐Caryolanediol	0.14 ± 0.01^*^	—
65	1823	Pentadecanoic acid	—	0.01 ± 0.00^§^
66	1845	Phytone	—	0.02 ± 0.00^§^
67	1861	(Z,Z)‐Farnesyl acetone	0.20 ± 0.01^*^	—
68	1921	Hexadecanoic acid, methyl ester	0.01 ± 0.00	0.01 ± 0.00
69	1943	Phytol	—	0.02 ± 0.00^§^
70	1946	Isophytol	—	2.38 ± 0.12^§^
71	1957	Geranyl benzoate	—	0.02 ± 0.00^§^
72	1960	Hexadecanoic acid	1.10 ± 0.08^*^	0.06 ± 0.00
73	2013	α‐Springen	0.02 ± 0.00^*^	—
74	2015	2,6,11,15‐Tetramethyl‐hexadeca‐2,6,8,10,14‐pentaene	0.01 ± 0.00^*^	—
75	2018	(6E,10Z)‐pseudo phytol	0.54 ± 0.02^*^	1.03 ± 0.04
76	2038	Heptadecanoic acid	0.01 ± 0.00^*^	0.03 ± 0.00
77	2085	Methyl linoleate	0.01 ± 0.00	0.01 ± 0.00
78	2133	Linoleic acid	0.87 ± 0.04	0.78 ± 0.03
79	2187	Octadecanoic acid	0.23 ± 0.01^*^	0.49 ± 0.02
80	2305	Δ^9^‐Tetrahydrocannabivarin	0.03 ± 0.00^*^	0.01 ± 0.00
81	2342	Cannabicyclol	0.50 ± 0.03^*^	1.27 ± 0.03
82	2435	Cannabielsoin	1.65 ± 0.06^*^	0.11 ± 0.00
83	2441	Cannabidiol	74.26 ± 1.24^*^	78.66 ± 1.54
84	2486	Cannabichromene	0.25 ± 0.01^*^	0.04 ± 0.00
85	2446	Δ^8^‐Tetrahydrocannabinol	0.20 ± 0.01^*^	0.16 ± 0.00
86	2492	Δ^9^‐Tetrahydrocannabinol	3.62 ± 0.24	3.91 ± 0.21
87	2548	Cannabigerol	1.12 ± 0.05^*^	0.78 ± 0.02
88	2558	Cannabinol	0.93 ± 0.04^*^	1.44 ± 0.03
89	2682	Heptadecane, 9‐octyl	0.17 ± 0.01	0.19 ± 0.01
90	2702	Heptacosane	1.20 ± 0.07^*^	1.42 ± 0.01
91	2805	Octacosane	0.31 ± 0.02	0.29 ± 0.01
92	2808	Squalene	0.23 ± 0.01^*^	0.31 ± 0.02
93	2900	Nonacosane	6.07 ± 0.24	5.46 ± 0.21
94	3003	Triacontane	0.35 ± 0.02^*^	0.29 ± 0.01
95	3039	Heneicosane, 11‐decyl‐	—	0.19 ± 0.01^§^
96	3398	Triacontyl acetate	0.13 ± 0.00^*^	—
Cannabinoids			82.56	86.38
Monoterpenes			0.20	0.02
Oxygenated monoterpenes			0.17	—
Sesquiterpenes			2.97	0.04
Oxygenated sesquiterpenes			1.93	0.03
Alkanes			7.99	7.71
Fatty acids			2.64	1.67
Alcohols			0.73	3.55
Others			0.81	0.60

Abbreviations: #, Elution order on HP‐5‐MS column; — = not detected; *t* = traces, ≤0.01%.

^a^ Kovats retention index related to an alkane standard mix C_7_‐C_40_ on HP‐5MS column.

^*^ *p* < .001 versus CFHE2.

^§^ *p* < .001 versus CFHE1.

Cannabinoids represent the most abundant compounds, and within this class, CBD has the highest mean area percentage in both extracts (74.26% and 78.66%). However, a slight but statistically significant difference in the relative abundance of cannabinoids (82.56% and 86.38% in CFHE1 and CFHE2, respectively) was recorded. This is mainly attributable to the loss of sesquiterpenes (2.97% vs. 0.04%, in CFHE1 and CFHE2, respectively) and oxygenated sesquiterpenes (1.93% vs. 0.03%, in CFHE1 and CFHE2, respectively) in CFHE2 during the hydrodistillation process.

Since acid cannabinoids are thermolabile, it is impossible to distinguish between acid and neutral forms following a GC–MS analysis, as they are immediately decarboxylated due to the high injector temperature (Smeriglio et al., 2018). Considering this, a reversed‐phase liquid chromatography coupled with diode array detection and electrospray ion trap tandem mass spectrometry (RP‐LC‐DAD‐ESI‐MS/MS) analysis was carried out in order to identify the native phytocannabinoid profile of the two investigated extracts.

**FIGURE 1:**
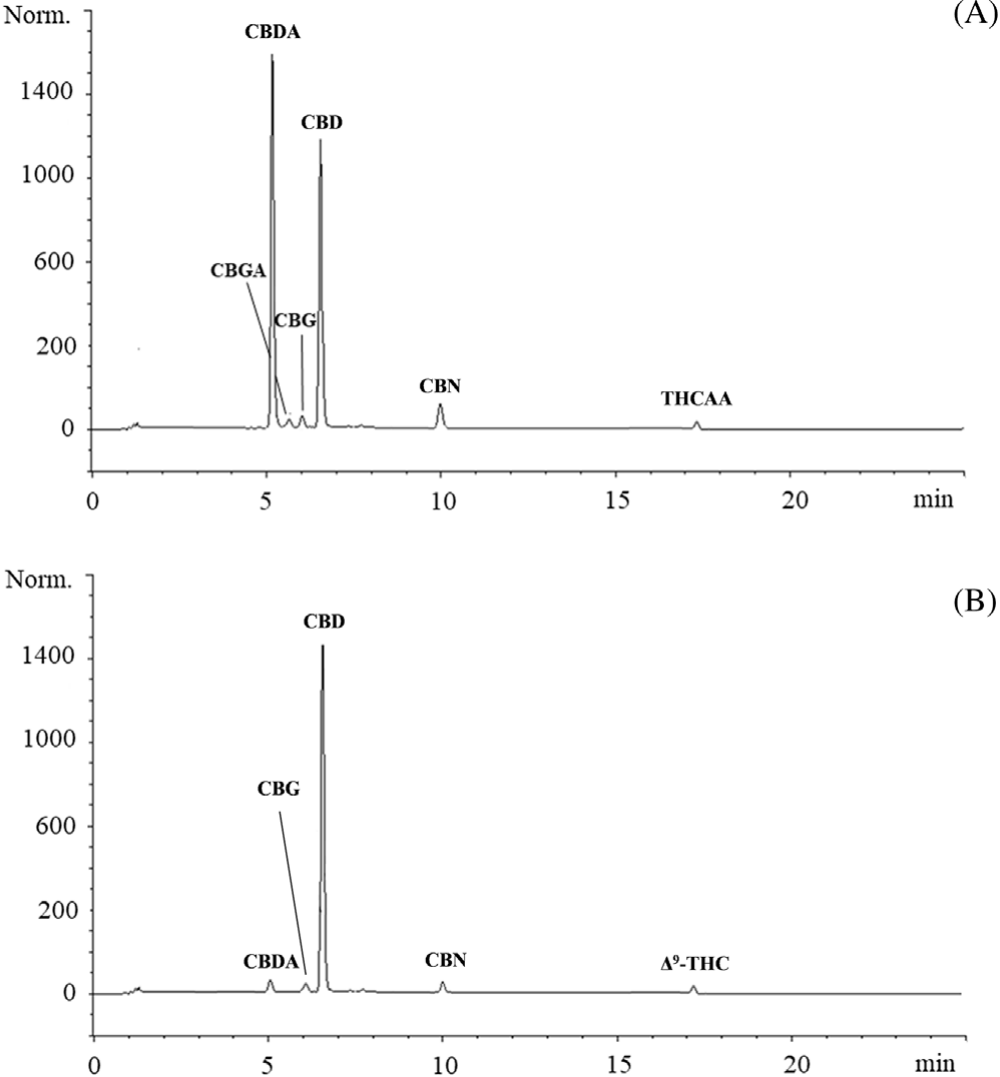
Representative liquid chromatography (LC)‐DAD chromatograms acquired at 220 nm reporting the native phytocannabinoids profile of CFHE1 (a) and CFHE2 (b)

A high content of phytocannabinoids was detected in both extracts with a predominance of the acid and neutral cannabinoids in CFHE1 and CFHE2, respectively (Figure 1).

CBD (23,512.07 mg/100 g DE) was the most abundant cannabinoid identified into CFHE1, followed by its acid form CBDA (14,653.45 mg/100 g DE), CBG (364.15 mg/100 g DE), cannabigerolic acid (CBGA, 286.38 mg/100 g DE), CBN (231.57 mg/100 g DE), and THCAA (34.60 mg/100 g DE; Figure 1 and Table 2).

**TABLE 2:** Native phytocannabinoid profile of hexane extracts (CFHE1 and CFHE2) of *Cannabis sativa* var. *fibrante* by RP‐LC‐DAD‐ESI‐MS/MS analysis. Results were expressed as mg/100 g DE and represent the mean ± SD of three independent experiments in triplicate (*n* = 3)

				mg/100 g DE	
Acids	RT (min)	MS (m/z) [M‐H]^−^	MS/MS (*m/z*) [M‐H]^−^	CFHE1	CFHE2
CBDA	5.243	357	339, 245	14,653.45 ± 10.88^*^	122.90 ± 1.24
CBGA	5.742	359	341, 359	286.38 ± 2.54^*^	≤ LOD
CBNA	7.570	353	309, 279	≤ LOD	≤ LOD
THCAA	17.385	357	313, 245	34.60 ± 0.67^*^	≤ LOD

Neutrals	RT (min)	MS (*m/z*) [M‐H]^+^	MS/MS (*m/z*) [M‐H]^+^	mg/100 g DE	
CBG	6.131	317	207, 233	364.15 ± 1.88^*^	285.40 ± 1.08
CBD	6.498	315	259, 233	23,512.07 ± 58.44^*^	27,556.12 ± 27.55
CBN	10.077	311	223, 43	231.57 ± 5.62^*^	113.56 ± 1.85
Δ^9^‐THC	17.269	315	245, 193	≤ LOD^*^	20.25 ± 0.28

Abbreviations: DE, dry extract; RT, retention time.

^*^ *p* < .001 versus CFHE2.

Following hydrodistillation, there is a strong depletion of CBDA, resulting in an increase in its neutral form, CBD (27,556.12 mg/100 g DE), which was the most abundant phytocannabinoid identified into CFHE2, followed by CBG (285.40 mg/100 g DE), CBDA (122.90 mg/100 g DE), CBN (113.56 mg/100 g DE), and Δ^9^‐THC (20.25 mg/100 g DE), decarboxylation product of THCAA (Figure 1 and Table 2).

The phytochemical profile of the investigated extracts reflects that previously found for other hemp extracts, in which neutral cannabinoids CBD and CBG and their corresponding acid forms (CBDA and CBGA) were the predominant cannabinoids (Brighenti, Pellati, Steinbach, Maran, & Benvenuti, 2017; Smeriglio et al., 2018), followed by CBN and Δ^9^‐THC (McPartland & Russo, 2001).

However, although cannabinoids represent the most abundant compounds in *Cannabis* extracts, terpenes play also a pivotal role, synergizing the phytocannabinoids' activity and enhancing the several health effects of *Cannabis* (Sommano, Chittasupho, Ruksiriwanich, & Jantrawut, 2020).

The terpene profile of CFHE1 reflects that found previously in the essential oil of *C. sativa* var. *fibrante*, in which β‐caryophyllene and caryophyllene oxide were the most representative compounds (Smeriglio et al., 2020). These results are also in accordance with Gulluni et al. (2018), who found caryophyllene derivatives as the most abundant sesquiterpenes (21.74%) in *Cannabis sativa* L. var. monoica, and with Zengin et al. (2018), who showed that the most abundant compounds of *C. sativa* cv. Futura 75 EO belong to the class of sesquiterpenes (67% of the total identified compounds), with β‐caryophyllene as the most abundant compound (28%). However, recently, a high variability between the phytochemical profiles of different fiber‐type varieties of *C. sativa*, was observed (Iseppi et al., 2019). Indeed, although the most abundant compounds detected are always the same, the relative abundance of the different classes of terpenes as well as of phytocannabinoids is quite different among the *Cannabis* varieties, cultivar, or biotypes (Smeriglio et al., 2020).

### 3.2 Determination of antioxidant properties

**FIGURE 2:**
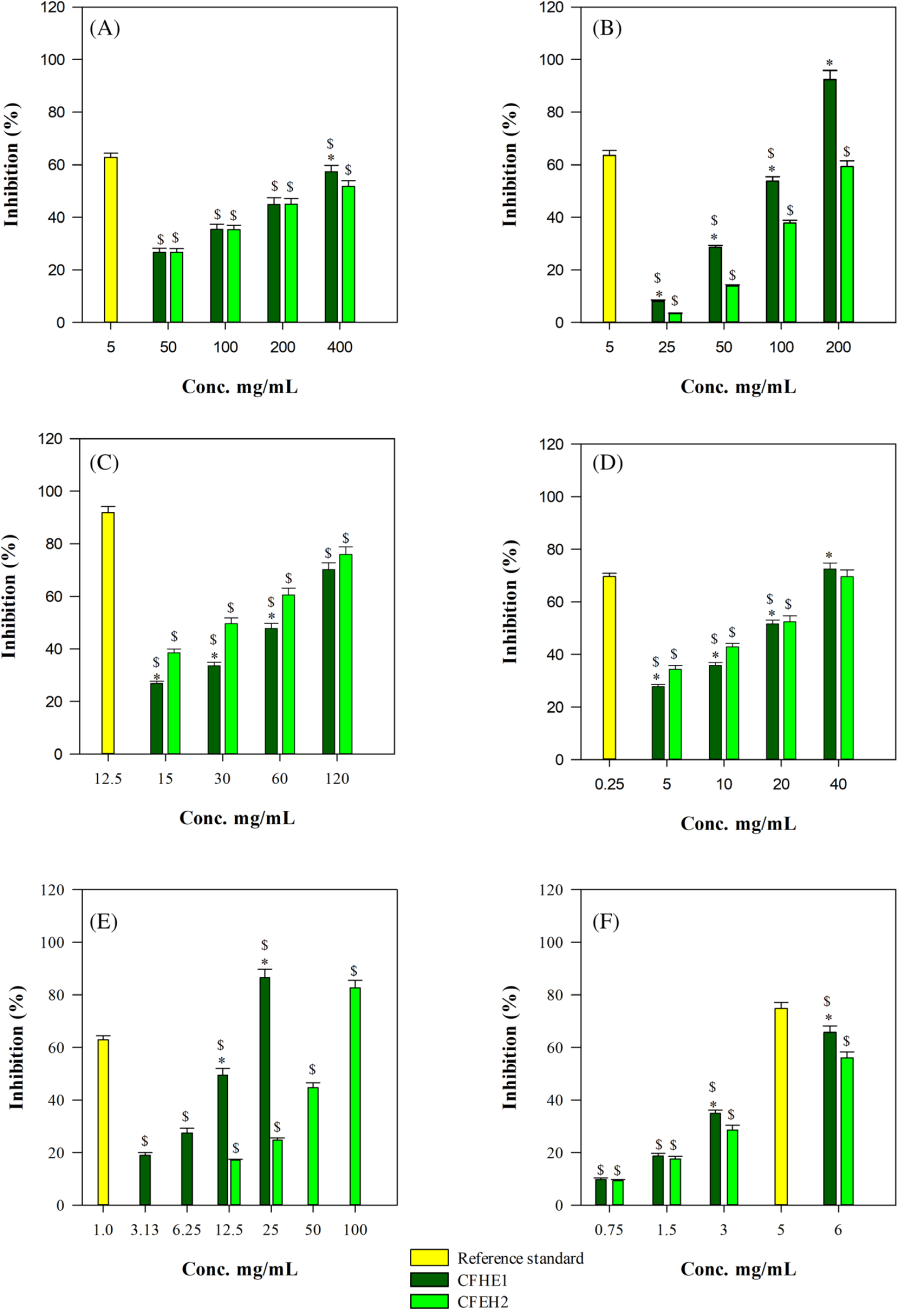
Antioxidant and free radical‐scavenging activity of CFHE1 and CFHE2 toward (a) 2,2‐diphenyl‐1‐picrylhydrazyl (DPPH), (b) ferric reducing antioxidant power (FRAP), (c) Ferrozine, (d) β‐carotene bleaching, (e) oxygen radical absorbance capacity (ORAC) and (f) trolox equivalent antioxidant capacity (TEAC) assay. ^*^ *p* < .001 versus CFHE2; ^$^ *p* < .001 versus reference standard: butylhydroxytoluene (BHT) for β‐carotene bleaching assay; ethylenediaminetetraacetic acid (EDTA) for ferrozine assay; trolox for DPPH, FRAP, TEAC and ORAC assays

Antioxidant and free‐radical scavenging potential of *Cannabis* extracts was evaluated by several in vitro cell‐free assays based on different reaction mechanisms and charged radicals. Both CFHE1 and CFHE2 showed remarkable and concentration‐dependent antioxidant and free‐radical scavenging activity (Figure 2).

CFHE1 showed the following order of potency: TEAC > ORAC > β‐carotene bleaching > Iron‐chelating activity > FRAP > DPPH. On the contrary, CFHE2 showed the following order of potency: TEAC > β‐carotene bleaching > Iron‐chelating activity > ORAC > FRAP > DPPH (Table 3).

**TABLE 3:** Antioxidant and free‐radical scavenging activity of CFHE1 and CFHE2 in comparison with reference compounds. Results were expressed as mean half‐inhibitory concentration (IC_50_ μg/ml) with confident limits (CL) at 95% of three independent experiments in triplicate (*n* = 3)

Assay	CFHE1	CFHE2	Reference compound^a^
TEAC	4.17 (3.40–5.11)^ψ^ ^,^ ^§^	5.65 (4.46–7.17)^§^	2.93 (1.80–4.38)
ORAC	12.51 (6.82–22.93)^ψ^ ^,^ ^§^	56.73 (25.65–125.44)^§^	0.67 (0.31–1.22)
β‐Carotene bleaching	18.05 (14.20–22.96)^ψ^ ^,^ ^§^	17.28 (12.51–23.88)^§^	0.18 (0.09–0.36)
Iron‐chelating activity	63.43 (49.12–81.91) ^ψ,^ ^§^	33.02 (24.64–44.26)^§^	6.59 (5.21–8.04)
FRAP	80.21 (43.56–147.69)^ψ^ ^,^ ^§^	144.86 (118.12–177.65)^§^	3.73 (1.68–7.59)
DPPH	254.10 (177.50–363.75)^ψ^ ^,^ ^§^	317.23 (207.35–485.34)^§^	3.82 (1.12–5.38)

^a^ Trolox for trolox equivalent antioxidant capacity (TEAC), oxygen radical absorbance capacity (ORAC), ferric reducing antioxidant power (FRAP) and 2,2‐diphenyl‐1‐picrylhydrazyl (DPPH) assays; butylhydroxytoluene (BHT) for β‐carotene bleaching assay; ethylenediaminetetraacetic acid (EDTA) for iron‐chelating activity.

^ψ^ *p* < .001 versus CFHE2.

^§^ *p* < .001 versus reference compound.

CFHE1 showed the strongest and statistically significant (*p* < .001) antioxidant activity in all tests carried out in comparison with the CFHE2, with the exception of the β‐carotene bleaching and iron‐chelating activity assays (Table 3). Moreover, both extracts showed statistically significant results (*p* < .001) with respect to the reference compounds (Table 3).

The remarkable antioxidant activity found for both CFHE1 and CFHE2 could be justified by the conspicuous presence of bioactive molecules, in particular phytocannabinoids and terpenes. Recently it has been demonstrated, by several in vitro and in vivo studies on *Cannabis* extracts, that these are the main class of bioactive compounds responsible of the antioxidant activity of *Cannabis* plant complexes (Nuutinen, 2018; Pellati et al., 2018). CDB, for its countless biological properties, represents the most investigated non‐psychotropic cannabinoids from a pharmacological point of view (Appendino, Chianese, & Taglialatela‐Scafati, 2011; Atalay, Jarocka‐Karpowicz, & Skrzydlewska, 2019; Campos, Fogaça, Sonego, & Guimarães, 2016; Hartsel, Eades, Hickory, & Makriyannis, 2016; Izzo, Borrelli, Capasso, Di Marzo, & Mechoulam, 2009). However, terpenes can also exert a pivotal role (Nafis et al., 2019). It is well known that monoterpenes generally possess the strong antioxidant activity, following by oxygenated monoterpenes, sesquiterpenes, and oxygenated sesquiterpenes (Smeriglio et al., 2020). This could justify the strongest antioxidant activity of CFHE1 in comparison with CFHE2, since it is the richest source of these volatile bioactive compounds, which pass into the essential oil after hydrodistillation. However, it is well known that neutral phytocannabinoids, particularly CBD, possess a marked antioxidant activity by reducing the lipid and protein modifications (direct antioxidant activity) as well as by the activation, antagonization or inhibition of cannabinoid (CB1 and CB2), ionotropic (TRP) and nuclear (PPAR) receptors (indirect antioxidant activity) (Atalay et al., 2019), and this could explain the sometimes‐fluctuating behavior of the two extracts investigated in the present study. Although CFHE2 is poor as regards the terpene component, it is, instead, very rich in neutral cannabinoids and in particular in CBD. Moreover, as previously observed, *Cannabis* plant complexes generally exert the strongest activity in comparison with the most representative isolated compounds, highlighting a possible synergistic mechanisms between the different classes of compounds as previously observed (Nafis et al., 2019; Smeriglio et al., 2018; Smeriglio et al., 2020).

### 3.3 Antimicrobial properties

A preliminary antimicrobial screening against the Gram‐negative bacteria *Pseudomonas aeruginosa* ATCC 9027 and *E. coli* ATCC 10536 highlighted that both extracts did not show any activity against *P. aeruginosa*, whereas a weak effect was detected against *E. coli*, with MIC values of 1,250 to 2,500 μg/ml for CFHE2 and CFHE1, respectively (Table 4).

**TABLE 4:** Minimum inhibitory concentration (MIC) and minimum fungicidal concentration (MFC) of CFHE1, CFHE2 and reference compounds against the Gram‐negative *Pseudomonas aeruginosa* ATCC 9027 and *Escherichia coli* ATCC 10536 bacteria, and the yeast *Candida albicans* ATCC 10231. Results (μg/ml) were expressed as mean ± S.D. of three independent experiments in triplicate (*n* = 3)

	CFHE1	CFHE2	Reference compound
MIC (μg/ml)			
Gram‐negative			Tobramycin
*P. aeruginosa* ATCC 9027	NA	NA	0.23 ± 0.01
*E. coli* ATCC 10536	2500^*^	1250^*^	0.48 ± 0.02
MFC (μg/ml)			
Yeast			Caspofungin
*C. albicans* ATCC 10231	NA	NA	0.061 ± 0.00

Abbreviation: NA, not active.

^*^ *p* < .001 versus tobramycin.

In our recent investigation on the antioxidant and antimicrobial activity of two standardized extracts from *C. sativa* L. (Muscarà et al., 2021), we reported no antimicrobial effect against any of the Gram‐negative strains tested. Here, *E. coli* was slightly sensitive to both extracts, indicating a potential therapeutic tool against Gram‐negative bacteria. It is widely accepted that Gram‐negative bacteria are more resistant to natural extracts compared to the Gram‐positive strains based on the differences in cell wall composition. In our previous work (Mandalari et al., 2007), bergamot fractions obtained from the *Citrus* fruit processing industry, were found to be active against all the Gram‐negative bacteria tested, which included *E. coli*, *Pseudomonas putida*, *Salmonella enterica*. In agreement with our previous investigation on *C. sativa* L. (Muscarà et al., 2021), no antifungal potential was observed against the yeast *C. albicans* ATCC 10231. Both CFHE1 and CFHE2 showed a strong antibacterial activity against *S. aureus* ATCC 6538, with very interesting MIC (4.88 μg/ml for both CFHE1 and CFHE2) and MBC (4.88 μg/ml and 19.53 μg/ml for CFHE1 and CFHE2, respectively) values (Table 5).

**TABLE 5:** Minimum inhibitory concentration (MIC), MIC_50_, MIC_90_, and minimum bactericidal concentration (MBC) (μg/ml) of CChHE1 and CChHE2 against *Staphylococcus aureus* ATCC 6538 and 19 MRSA clinical strains of *Staphylococcus aureus* obtained by three independent experiments in triplicate (*n* = 3)

	MIC				MBC		
	CFHE1		CFHE2		CChHE1	CChHE2	Vancomycin
*S. aureus* ATCC 6538	4.88		4.88		4.88^§^	19.53^§^	0.32–0.64

	MIC_50_		MIC_90_		MBC		
	CFHE1	CFHE2	CFHE1	CFHE2	CFHE1	CChHE2	Vancomycin
MRSA clinical strains	1.22–4.88	2.44–4.88	9.77	9.77	4.88–78.13^§^	4.88–78.13^§^	0.32–0.64

Abbreviation: MRSA, methicillin‐resistant *S. aureus*.

^§^ *p* < .001 versus vancomycin.

Based on these data, our attention was focused on clinical methicillin‐resistant strains of *S. aureus*. Both extracts showed bacteriostatic and bactericidal activity against the clinical strains, with MIC_50_ values between 1.22 and 4.88 μg/ml and MIC_90_ of 9.77 μg/ml. The MBC values ranged between 4.88 and 78.13 μg/ml, with no significant differences between the two extracts (Table 5). *S. aureus* currently represents a major threat to public health given the range of causing infections, both localized and systemic, and the selection of multidrug resistant strains. Therefore, the search of novel sources of natural antimicrobials could be promising for the treatment of topical infections. A recent study by Žitek et al. (2020) reported on the anticancer and antimicrobial ability of a combination of ginger and cannabis extracts used different ratios: the results demonstrated a bacteriostatic effect against *S. aureus*, *E. coli*, and *C. albicans* in *Cannabis*‐dominated ratios. The combination of *Cannabis* extracts with either natural compounds or traditional antibiotics may help strengthen potential synergistic interactions to overcome antibiotic resistance.

In a recent investigation, the antimicrobial effect of *C. sativa* Futura 75 was evaluated in vitro against foodborne pathogens, and on food against natural bacterial groups of minced meat stored for 8 days at 4°C (Pasquali et al., 2020). In agreement with our data, the results showed an in vitro effect against *S. aureus* at a concentration between 0.017 and 0.15 mg/ml. However, no effect was observed on Gram‐negative bacteria including *E. coli*.

Based on these findings, it is possible to postulate the use of these *Cannabis* extracts as natural antimicrobials with bactericidal effect, particularly against Gram‐positive bacterial infections.

## 4 CONCLUSIONS

This is the first study investigating the native phytocannabinoid and terpenic profile as well as the antioxidant and antimicrobial activity of standardized extracts from flowering tops of *Cannabis sativa* L. var. *fibrante* CBD‐chemotype as such and after hydrodistillation. Moreover, this is the first study evaluating the activity of these *Cannabis* extracts on virulent strains of *Staphylococcus*, which have always been of concern for human health, particularly MRSA strains, responsible for many nosocomial infections.

The strong antioxidant and free‐radical scavenging activity found in both hexane extracts (pre‐ and post‐hydrodistillation) allow to postulate that the compounds mainly responsible of the antioxidant power are cannabinoids and in particular their neutral forms, although a synergistic effect due to the presence of minor compounds, in particular terpenes, cannot be excluded. CFHE1 proved to be the most powerful extract against the Gram‐positive *S. aureus* probably due to the presence of terpenes in addition to cannabinoids, although CFHE2 maintains an interesting antibacterial activity toward both ATCC and clinical MRSA strains.

Considering this and according to the antioxidant results, cannabinoids seem to exert a pivotal role in the antimicrobial activity, shedding light on a promising potential use of these standardized hexane extracts as antibacterial agents for the treatment of *S. aureus* infections.

However, these are preliminary data needing further investigation, both regarding the antibacterial activity against *S. aureus* and the safety profile of these extracts. One of the virulence factor of *S. aureus* is given by its ability to form biofilms both on abiotic and biotic surfaces. Therefore, it becomes essential to determine the effectiveness of these extracts even in these conditions, alone or in combination with synthetic antibiotics, trying to formulate a possible therapeutic application, which allows an appropriate study of their toxicological profile.

## CONFLICT OF INTEREST

The authors declare that there is no conflict of interest.

## Data Availability

The data that support the findings of this study are available from the corresponding author upon reasonable request.
